# HLH‐30‐dependent rewiring of metabolism during starvation in *C*. *elegans*

**DOI:** 10.1111/acel.13342

**Published:** 2021-03-16

**Authors:** Kathrine B. Dall, Jesper F. Havelund, Eva B. Harvald, Michael Witting, Nils J. Færgeman

**Affiliations:** ^1^ Department of Biochemistry and Molecular Biology Villum Center for Bioanalytical Sciences University of Southern Denmark Odense M Denmark; ^2^ Research Unit Analytical BioGeoChemistry Helmholtz Zentrum München Neuherberg Germany; ^3^ Metabolomics and Proteomics Core Helmholtz Zentrum München Neuherberg Germany; ^4^ Chair of Analytical Food Chemistry Technische Universität München Freising Germany

**Keywords:** aging, *Caenorhabditiselegans*, lipidomics, metabolomics, mitochondria, peroxisome, starvation, β‐oxidation

## Abstract

One of the most fundamental challenges for all living organisms is to sense and respond to alternating nutritional conditions in order to adapt their metabolism and physiology to promote survival and achieve balanced growth. Here, we applied metabolomics and lipidomics to examine temporal regulation of metabolism during starvation in wild‐type *Caenorhabditis elegans* and in animals lacking the transcription factor HLH‐30. Our findings show for the first time that starvation alters the abundance of hundreds of metabolites and lipid species in a temporal‐ and HLH‐30‐dependent manner. We demonstrate that premature death of *hlh*‐*30* animals under starvation can be prevented by supplementation of exogenous fatty acids, and that HLH‐30 is required for complete oxidation of long‐chain fatty acids. We further show that RNAi‐mediated knockdown of the gene encoding carnitine palmitoyl transferase I (*cpt*‐*1*) only impairs survival of wild‐type animals and not of *hlh*‐*30* animals. Strikingly, we also find that compromised generation of peroxisomes by *prx*‐*5* knockdown renders *hlh*‐*30* animals hypersensitive to starvation, which cannot be rescued by supplementation of exogenous fatty acids. Collectively, our observations show that mitochondrial functions are compromised in *hlh*‐*30* animals and that *hlh*‐*30* animals rewire their metabolism to largely depend on functional peroxisomes during starvation, underlining the importance of metabolic plasticity to maintain survival.

## Graphical abstract

We have applied a combinatorial metabolomics and lipidomics approach to examine temporal regulation of metabolism during starvation and how HLH‐30 regulates metabolism during starvation in *C. elegans*. We find that starvation increases the abundance of cardiolipins and acyl‐carnitines and enhances fatty acid oxidation in an HLH‐30‐dependent manner. Loss of HLH‐30 renders *C. elegans* strictly dependent of peroxisomal biogenesis during starvation to maintain survival. Our findings underlines the importance of metabolic plasticity to respond and adapt to varying nutritional conditions.

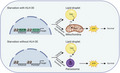

## 1 INTRODUCTION

The ability to regulate metabolism in response to changes in nutrient availability is an evolutionarily conserved mechanism ranging from bacteria to humans. Regulating metabolism by coordinating anabolic and catabolic pathways to sustain metabolic homeostasis ensures prolonged survival during periods of nutrient scarcity. However, rewiring of energy metabolism is a complex and dynamic process encompassing many transcriptional and post‐transcriptional regulators. One of the fundamental mechanisms promoting survival in response to starvation is the use of energy stores, for example, via breakdown of lipids. Fatty acids are mobilized from lipid droplets in adipocytes for mitochondrial β‐oxidation through either lipolysis or lipophagy; pathways that have both been reported to be crucial for surviving starvation (Martin & Parton, 2006). A major regulator of lipid metabolism during starvation is the conserved basic helix‐loop‐helix transcription factor HLH‐30 in *Caenorhabditis elegans* (*C. elegans*), an ortholog of the mammalian transcription factor EB (TFEB; Lapierre et al., 2013). HLH‐30/TFEB regulates the expression of genes belonging to the coordinated lysosomal expression and regulation (CLEAR) network, which are involved in autophagosome formation, lysosomal biogenesis, lipase function, and fatty acid degradation (Martina et al., 2014; Palmieri et al., 2011; Settembre et al., 2013). HLH‐30/TFEB is also a transcription factor‐mediating resistance to several stressors besides starvation, including oxidative stress, heat stress, and host defense against pathogen infection (Lin et al., 2018; Visvikis et al., 2014). Additionally, removal of HLH‐30/TFEB impairs the longevity of several long‐lived *C. elegans* mutants (Lapierre et al., 2013) and entry into adult reproductive diapause (Gerisch et al., 2020), and the *hlh*‐*30* mutant itself dies prematurely during starvation (Harvald et al., 2017; O'Rourke & Ruvkun, 2013).

In the present study, we have successfully applied a combinatorial metabolomics and lipidomics approach to examine temporal regulation of metabolism during starvation and how HLH‐30 regulates metabolism during starvation in *C. elegans*. Specifically, we find that starvation induces significant and specific changes in the metabolome and in the lipidome of *C. elegans*. In particular, we find that starvation induces both long‐chain acyl‐carnitine and cardiolipin levels in wild‐type animals, in accordance with enhanced mitochondrial metabolism. Accordingly, we find that starvation induces oxidation of oleic acid. Markedly, induction of cardiolipin and acyl‐carnitine levels and oxidation of oleic acid upon starvation are completely absent in *hlh*‐*30* animals, arguing that HLH‐30 is required for induction of mitochondrial β‐oxidation during starvation. Interestingly, we find that impaired generation of peroxisomes induces premature death of *hlh*‐*30* animals upon starvation, which cannot be rescued by supplementation of exogenous fatty acids. Collectively, we show for the first time, that functional loss of HLH‐30 renders *C. elegans* highly dependent on peroxisomal degradation of fatty acids to survive starvation. Our observations substantiate the importance of metabolic plasticity in order to survive periods of nutrient scarcity.

## 2 RESULTS

### 2.1 Starvation induced metabolic and lipidomic re‐arrangement enhances mitochondrial function in *C. elegans*

**FIGURE 1:**
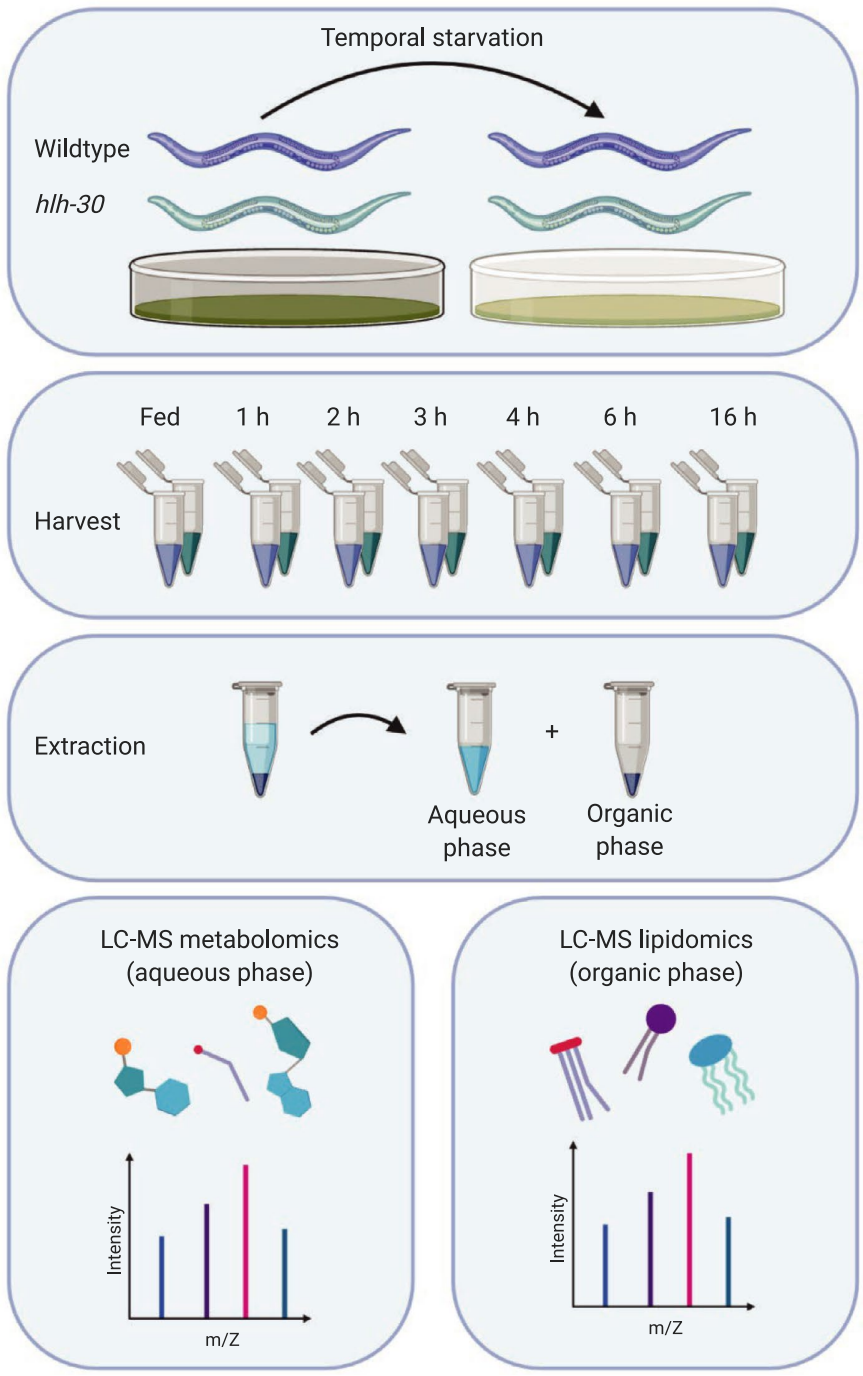
Experimental workflow for combined metabolomics and lipidomics of the starvation response in *C. elegans*. Wild‐type *C. elegans* and the *hlh*‐*30* mutant were included in the study. Starvation was induced by transferring animals to plates containing no bacteria. Worms were starved for 1, 2, 3, 4, 6, and 16 h of starvation, prior to harvesting and extraction of metabolites and lipids. The upper aqueous phase was analyzed using LC–MS‐based metabolomics, and the lower organic phase was analyzed using LC–MS‐based lipidomics

To identify the metabolic response to starvation, we analyzed the temporal response to starvation in *C. elegans* across a 16 h starvation time course at the mid‐L4 stage by metabolomics and lipidomics (Figure 1). As previously (Harvald et al., 2017), we analyzed multiple time points within the first 6 h to interrogate the early starvation responses with high resolution. We harvested animals in biological triplicate at each of the 7 time points for extraction of metabolites and lipids, respectively, and for subsequent analyses by MS‐based metabolomics and lipidomics (Figure 1). To optimize lipid extraction from *C. elegans*, we applied different commonly used lipid extraction methods and performed lipid profiling in positive ionization mode. By using the BUME extraction (Lofgren et al., 2012), we detected 4.963 different molecular features in the apolar phase, while we detected 4819, 4249, 5172, and 5082 molecular features when using Bligh and Dyer (Bligh & Dyer, 1959), Folch (Folch et al., 1957), MMC (Pellegrino et al., 2014), and MTBE (Matyash et al., 2008) extraction methods, respectively (Figure S1). 4143 features were commonly detected in all tested extraction methods (Figure S1). Although the MTBE extraction yielded the most features, we also found that this method showed the highest extraction variability. In contrast, the Folch extraction showed not only the lowest variability, but also the lowest number of features (Figure S1). Based on the overall performance, the BUME extraction method not only showed a high number of features with a low variability, but also provided a polar phase for LC–MS metabolomic analyses of polar metabolites. Furthermore, it showed better recovery of polar lipids such as lysolipids compared with the other methods. Applying the BUME extraction and lipid profiling to our samples, 4063 lipid features in positive and 2258 in negative ionization mode remained after normalization and filtering (detected in all QCs and RSD < 30%). Out of these, 2068 were putatively annotated on the MS^1^ level and 427 on the MS^2^ level in positive ionization mode, 955 and 118 in negative mode, respectively (Table S1).

**FIGURE 2:**
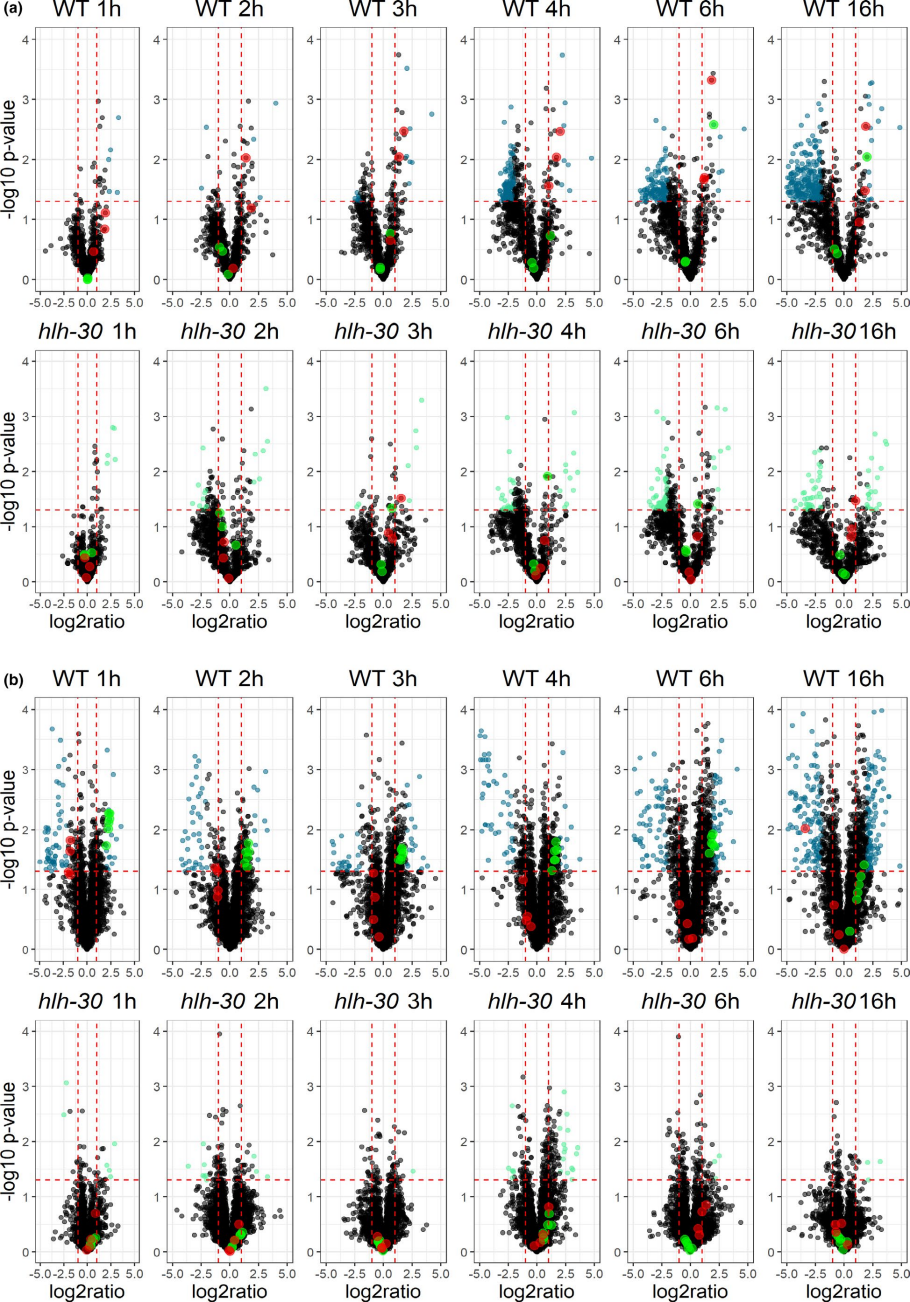
Metabolic and lipidomic changes induced by temporal starvation. (a) Volcano plot displaying changes in the metabolome in response to starvation for the wild‐type and *hlh*‐*30* mutant at each time point. Significant up‐ or downregulated metabolites in wild‐type animals are shown in blue, and in light green for the *hlh*‐*30* animals. Regulation of long‐chain acyl‐carnitines are shown in red and short‐chain acyl‐carnitines are shown in green for both wild‐type and the *hlh*‐*30* mutant. Only, metabolites detected in the positive mode are shown. (b) Volcano plot displaying changes in the lipidome in response to starvation for the wild‐type and *hlh*‐*30* animals at each time point. Significant up‐ or downregulated lipids in the wild type are shown in blue, and in light green for *hlh*‐*30* animals. Regulation of cardiolipins are shown in green and N‐acylethanolamines are shown in red for both wild‐type and *hlh*‐*30* animals. Only, lipid species detected in the positive mode are shown. Metabolites and lipids with a *p*‐value <.05 and a fold‐change of >2 or <0.5 were considered to be significantly changed

**FIGURE 3:**
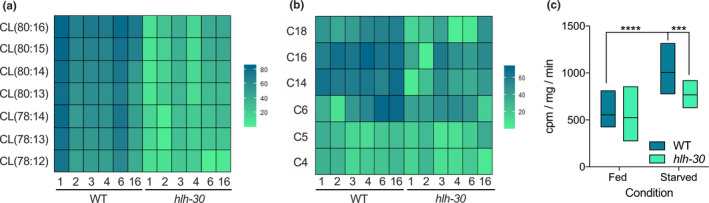
Acyl‐carnitine and cardiolipin regulation is disrupted in the *hlh*‐*30* mutant. (a) Heatmap illustrating the log_2_ fold‐change of specific long‐chain acyl‐carnitines at each starvation time point compared with fed condition for the wild‐type and *hlh*‐*30* mutant. (b) Heatmap illustrating the log_2_ fold‐change of specific cardiolipins at each starvation time point compared to fed condition for the wild‐type and *hlh*‐*30* mutant. Blue: upregulation, green: downregulation. (c) Oxidation of ^3^H‐labeled oleic acid is shown for wild‐type and *hlh*‐*30* animals in fed state and after 6 h of starvation ****p‐value <.0001, ***p‐value <.0009

We generated volcano plots to visualize to which extend starvation rewires the *C. elegans* metabolome across a 16 h starvation time course (Figures 2a and S2). This showed that short‐term starvation (1 and 2 h) only has subtle effects on the metabolome in wild‐type animals, while starvation for 3 h profoundly changes the metabolome (61 significantly altered metabolites in total in negative and positive modes; Table S1). Further, we found that the abundance of 289, 271, and 501 molecular features was significantly altered after 4, 6, and 16 h of starvation, respectively (Figures 2a and S2, and Table S1). During starvation, lipolysis of triacylglycerols plays an important role as an energy source in both mammals and in *C. elegans* (Buis et al., 2019; Lee et al., 2014; Martin & Parton, 2006; Murphy et al., 2019; Zaarur et al., 2019). Accordingly, the fatty acids released by lipolysis are subsequently activated to CoA‐esters and transported into the mitochondria by the carnitine‐shuttle system for degradation by β‐oxidation. Consistently, we observed that the level of long‐chain acyl‐carnitines massively increased already after one hour and remained elevated after 16 h of starvation in wild‐type animals, while levels of short‐chain acyl‐carnitines largely remained unchanged (Figures 2a and 3a).

By similar means, we visualized how the *C. elegans* lipidome alters across the starvation time course (Figures 2b and S3). The volcano plots clearly show that starvation also rewires the lipidome already after 1 h, as 79, and 99 lipid species were significantly up‐ or downregulated in positive and negative mode, respectively (Figures 2b and S3, and Table S1). Consistent with previous observations (Lucanic et al., 2011), we also found that the abundance of lipids like N‐acylethanolamines (NAEs) decreased in wild‐type animals across the starvation time course (Figure 2b). Changed lipids were grouped according to their profile across time. Based on this, lipids could be separated into species changing early within the first time points and later responders, for example, triacylglycerols show changes at 16 h of starvation. Moreover, among the upregulated lipid species we identified seven lipid species in wild‐type animals that significantly increased across all time points except after 16 h of starvation. Based on their elution profile and MS‐fragmentation pattern, we identified these lipid species to be cardiolipins (Figure S4). Cardiolipins are major phospholipids almost exclusively located in the inner mitochondrial membrane and required for mitochondrial morphology, mitochondrial membrane dynamics, and energy production not only in *C. elegans* but also in other eukaryotes (Sakamoto et al., 2012; Sustarsic et al., 2018). Collectively, these results show that starvation induces major re‐arrangements of both the metabolome and lipidome, and that mitochondrial functions are enhanced by starvation in wild‐type *C. elegans*.

### 2.2 Rewiring of lipid metabolism and induction of β‐oxidation upon starvation depend on HLH‐30 in *C. elegans*

Since the transcription factor HLH‐30 and its mammalian ortholog TFEB previously have been shown to serve crucial functions during starvation, dietary restriction, and autophagy in both *C. elegans* and in mammals (Harvald et al 2017; Lapierre et al., 2013; Murphy et al., 2019; O'Rourke & Ruvkun, 2013; Roczniak‐Ferguson et al., 2012; Settembre et al., 2013), this prompted us to examine how functional loss of HLH‐30 modulates the metabolome and the lipidome in response to starvation in *C. elegans*. In contrast to wild‐type animals, we only found a limited number of metabolites and lipid species that changed significantly in response to starvation in *hlh*‐*30* animals (Figure 2b and Table S1). Markedly, we found that the level of long‐chain acyl‐carnitines in *hlh*‐*30* animals remained largely unchanged in response to starvation compared to wild‐type animals (Figures 2a and 3a), arguing that fatty acid import into mitochondria is compromised. In keeping with this notion, we also found that cardiolipin levels in *hlh*‐*30* animals largely remained unchanged in response to starvation (Figures 2b and 3b). We, therefore, speculate that HLH‐30/TFEB is required for biogenesis or for the maintenance of functional mitochondria. Thus, to corroborate these observations, we assessed how functional loss of HLH‐30 affected fatty acid oxidation during starvation in *C. elegans*, by examining complete oxidation of ^3^H‐labeled oleic acid. During fed conditions, oxidation of oleic acid is similar in *hlh*‐*30* animals and in wild‐type animals. However, consistent with previous observations, oxidation of oleic acid increased significantly after six hours of starvation in wild‐type animals, while it remained unchanged in *hlh*‐*30* animals (Figure 3c). This observation substantiates that HLH‐30 is required for induction of fatty acid oxidation during starvation and hence for metabolic adaptation during starvation. Interestingly, we did not observe any overt alterations in mitochondria morphology in *hlh*‐*30* animals by Mitotracker staining (results not shown), and collectively arguing that HLH‐30/TFEB is required for maintenance of functional mitochondria.

### 2.3 Fatty acid supplementation rescues premature death during starvation in the hlh‐30 mutant

**FIGURE 4:**
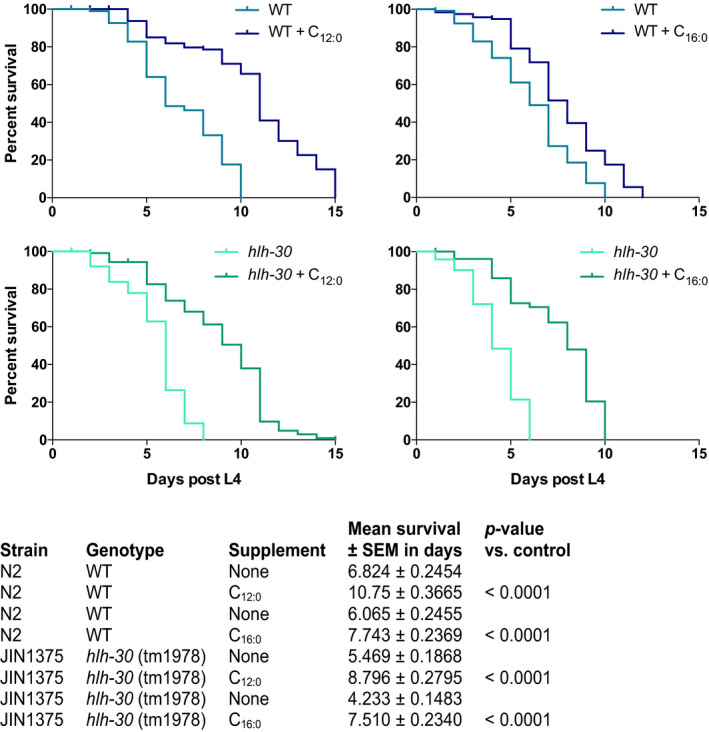
Exogenous fatty acid supplementation rescues premature death of the *hlh*‐*30* mutant during starvation. Survival‐span showing the effect of fatty acid supplementation on both wild‐type (blue) and *hlh*‐*30* mutant (green) survival in response to starvation. Starvation was induced at start L4 stage by transferring worms to empty plates. Both strains were supplemented with either vehicle, a medium‐chain fatty acid, lauric acid (C_12:0_) or a long‐chain fatty acid, palmitic acid (C_16:0_) throughout the experiment. Survival was monitored every day. Survival analysis was carried out using the Kaplan–Meier estimator and *p*‐value was calculated using log‐rank test in GraphPad Prism 6

TFEB, the mammalian ortholog of HLH‐30, has recently been found to be required for mitochondrial biogenesis, morphology, and functions in skeletal muscle in mice (Mansueto et al., 2017). Although loss of HLH‐30 functions does not affect mitochondria morphology in *C. elegans* (Murphy et al., 2019), the present observations show that HLH‐30 is required to support fundamental mitochondrial functions in *C. elegans* during limited nutritional conditions. Compared with wild‐type animals, *hlh*‐*30* animals die prematurely during starvation (Harvald et al., 2017). Since mobilization of fatty acids from intestinal lipid stores is required for *C. elegans* to withstand long‐term starvation (Buis et al., 2019), we, therefore, hypothesized that exogenous supplementation of medium‐chain fatty acids, which cross the mitochondrial membranes independent of the carnitine‐shuttle system, would rescue the premature death of *hlh*‐*30* animals. We, therefore, examined survival under starvation conditions by transferring animals to empty plates supplemented with either a medium‐chain (lauric acid, C_12:0_) or a long‐chain fatty acid (palmitic acid, C_16:0_). As previously, we found that *hlh*‐*30* animals die prematurely during starvation when compared to wild‐type animals. However, when supplemented with lauric acid both wild‐type and *hlh*‐*30* animals survived significantly longer during starvation when compared to un‐supplemented animals (Figure 4 and Table S2). In fact, *hlh*‐*30* animals were completely rescued to wild‐type levels by lauric acid. Supplementation with palmitic acid also extended the survival of wild‐type animals and surprisingly also of *hlh*‐*30* animals, however, not to the same extend as lauric acid (Figure 4 and Table S2). Notably, we found that uptake of palmitic acid in to both wild‐type and *hlh*‐*30* animals under starvation conditions is higher compared with uptake of lauric acid (results not shown), suggesting that the ability to rescue survival of *hlh*‐*30* animals is not due to increased uptake of lauric acid.

### 2.4 Disruption of the carnitine‐shuttle system impairs starvation survival of wild‐type animals

**FIGURE 5:**
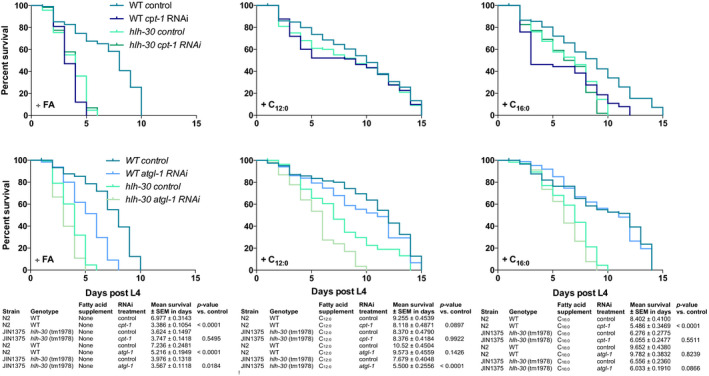
Survival of wild‐type animals is impaired by disruption of the carnitine‐shuttle. (a) Survival‐span showing the effect of RNAi‐mediated knockdown of *cpt*‐*1* on the survival of wild‐type and *hlh*‐*30* to mutant during starvation. Starvation was induced at start L4 stage by transferring worms to empty plates. Both strains were supplemented with either a medium‐chain fatty acid, lauric acid (C_12:0_) or a long‐chain fatty acid, palmitic acid (C_16:0_) throughout the experiment. Survival was monitored every day. (b) Survival‐span showing the effect of RNAi‐mediated knockdown of *atgl*‐*1* on the survival of wild‐type and *hlh*‐*30* to mutant during starvation. Starvation was induced at start L4 stage by transferring worms to empty plates. Both strains were supplemented with either a medium‐chain fatty acid, lauric acid (C_12:0_) or a long‐chain fatty acid, palmitic acid (C_16:0_) throughout the experiment. Survival was monitored every day. Survival analysis was carried out using the Kaplan–Meier estimator, and *p*‐value was calculated using log‐rank test in GraphPad Prism 6

Our findings support the notion that impaired β‐oxidation, caused by, for example, diminished mitochondrial import of long‐chain fatty acids, may be the underlying reason for the inability of the *hlh*‐*30* mutant to survive during starvation. By RNA‐sequencing Harvald et al. recently profiled the genome‐wide response to starvation (Harvald et al., 2017), and found that expression of genes encoding lipases needed for conventional lipolysis of triacylglycerols in lipid droplets (*atgl*‐*1*) or via lysosomal breakdown (*lipl*‐*2 to lipl*‐*4*) increased in wild‐type animals upon starvation but remained constant or decreased in *hlh*‐*30* animals during starvation (Figure S5). Similarly, the expression of genes encoding enzymes required for activation of fatty acids (*acs*‐*2*) and for active transport of fatty acids into the mitochondria (*cpt*‐*1*) is diminished in the mutant upon starvation (Figure S5). We, therefore, speculated that downregulation of *cpt*‐*1* expression would impair the ability of wild‐type animals to survive under starvation conditions. Intriguingly, we found that RNAi‐mediated knockdown of *cpt*‐*1* significantly impairs survival of wild‐type animals under starving conditions compared with the control animals, while *cpt*‐*1* knockdown had no effect on survival of *hlh*‐*30* animals (Figure 5 and Table S3). Markedly, lauric acid supplementation fully rescued the effects of *cpt*‐*1* knockdown in wild‐type animals and extended survival of *hlh*‐*30* animals to wild‐type levels independent of *cpt*‐*1* knockdown. Consistent with the notion that mitochondrial import of long‐chain fatty acids depends on a functional carnitine‐shuttle system, palmitic acid (C_16:0_) supplementation did not fully rescue survival of wild‐type control animals. Interestingly, palmitic acid supplementation rescued survival of *hlh*‐*30* animals under starvation conditions independent of *cpt*‐*1* knockdown, indicating that fatty acids can support survival during starvation by being channeled to other energy‐producing pathways than mitochondrial β‐oxidation.

ATGL‐1 mediates lipolysis of intestinal lipid stores during fasting in *C. elegans* (Lee et al., 2014). We, therefore, assessed whether the mobilization of stored lipids would affect survival during starvation. Expectedly, knockdown of *atgl*‐*1* in wild‐type animals significantly shortened survival compared with its control during starvation but had only minor effects on the survival‐span of *hlh*‐*30* animals (Figure 5b). Supplementation with lauric and palmitic acid both extended survival‐span of wild‐type and *hlh*‐*30* animals, yet only lauric acid extended survival‐span to wild‐type levels. All together, we interpret these observations that mobilization and mitochondrial import of fatty acids from lipid stores are crucial for surviving during starvation, however, only the latter is dependent on HLH‐30 in *C. elegans*.

### 2.5 Survival of the *hlh‐30* mutant during starvation is dependent on peroxisomal β‐oxidation

**FIGURE 6:**
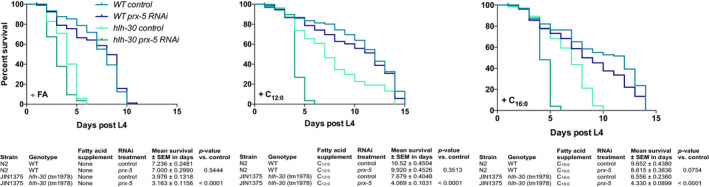
The ability of the *hlh*‐*30* mutant to survive starvation is dependent on peroxisomal β‐oxidation. Survival‐span showing the effect of RNAi‐mediated knockdown of *prx*‐*5* on the survival of wild‐type and *hlh*‐*30* to mutant during starvation. Starvation was induced at start L4 stage by transferring worms to empty plates. Both strains were supplemented with either a medium‐chain fatty acid, lauric acid (C_12:0_) or a long‐chain fatty acid, palmitic acid (C_16:0_) throughout the experiment. Survival was monitored every day. Survival analysis was carried out using the Kaplan–Meier estimator and *p*‐value was calculated using log‐rank test in GraphPad Prism 6

Since supplementation of palmitic acid also improved, survival of *hlh*‐*30* animals during starvation made us speculate whether *hlh*‐*30* animals compensate by using alternative metabolic pathways to generate sufficient energy to survive starvation. Besides mitochondria, peroxisomes are also capable of degrading fatty acids. Like mitochondrial β‐oxidation, peroxisomal β‐oxidation catalyzes chain shortening of acyl‐CoAs by four enzymatic steps yielding acetyl‐CoA. Despite that peroxisomal β‐oxidation in *C. elegans* is mostly known for oxidizing very long‐chain fatty acids (VLCFAs) and for the synthesis of ascarosides (Artyukhin et al., 2018), long‐ and medium‐chain saturated and unsaturated fatty acids can also serve as substrates for peroxisomal β‐oxidation (Poirier et al., 2006). The first step is catalyzed by the enzyme acyl‐CoA oxidase (ACOX), considered to be the main regulator of the flux through the pathway. Interestingly, expression of *acox* genes in *hlh*‐*30* animals is upregulated compared with wild‐type animals and sustain upregulated through starvation (Harvald et al., 2017; Figure S5). Therefore, since mitochondrial functions are compromised in *hlh*‐*30* animals, we speculated that they rewire their metabolism toward peroxisomes in order to survive starvation. Thus, by RNAi we knocked down *prx*‐*5* that encodes a peroxisomal assembly factor required for biogenesis of functional peroxisomes (Weir et al., 2017). Intriguingly, upon knockdown of *prx*‐*5*, survival of *hlh*‐*30* animals during starvation was dramatically decreased when compared to its control, while *prx*‐*5* knockdown in wild‐type animals did not affect survival during starvation (Figure 6 and Table S3). Moreover, neither lauric acid nor palmitic acid could rescue the effect of *prx*‐*5* knockdown, collectively implying that *hlh*‐*30* animals switch their metabolic program toward peroxisomes.

In conclusion, by using a systems‐wide analyses we have demonstrated how loss of the transcription factor HLH‐30 globally rewires metabolism in the nematode *C. elegans* during starvation. We found that animals lacking the transcription factor HLH‐30 fails to upregulate cardiolipin synthesis and β‐oxidation during starvation and compensate by upregulating peroxisomal functions. Collectively, our findings underline the importance of metabolic plasticity in order to adapt to varying nutritional conditions to ultimately promote survival.

## 3 DISCUSSION

This study provides a detailed metabolomic and lipidomic analyses characterizing temporal effects of acute starvation on metabolite levels in both wild‐type *C. elegans* and in animals lacking functional HLH‐30, an ortholog of the mammalian transcription factor TFEB. As recently shown (Harvald et al., 2017), the present study reveals that starvation massively rewires metabolism and lipid regulatory networks in a manner that is strongly dependent on the transcription factor HLH‐30. Here, we have detected 4063 lipid species belonging to more than 10 lipid classes and show that 996 distinct lipid species in positive and 792 in negative ionization mode change upon and during starvation. Similarly, among the 2147 metabolites that we have detected, the abundance of 775 and 738 metabolites change in at least one time point across the starvation series for both wild‐type and *hlh*‐*30* animals in positive and negative ionization mode, respectively. In particular, we find that distinct cardiolipin and acyl‐carnitine species change upon starvation but only in wild‐type animals and not in *hlh*‐*30* animals. Interestingly, these lipids are almost exclusively found or synthesized in mitochondria. Consistently, our findings show that both lipolysis of stored lipids and mitochondrial fatty acid oxidation are required for survival during starvation. Interestingly, supplementation of fatty acids rescues compromised survival of *hlh*‐*30* animals during starvation. Since fatty acid supplementation does not rescue longevity of *hlh*‐*30* animals after knockdown of the gene encoding the peroxisomal assemble factor PRX‐5, we speculate that HLH‐30/TFEB is required to generate and maintain functional mitochondria, and thus suggest that *hlh*‐*30* animals rewire metabolic activities toward peroxisomal activities to sustain survival during starvation. Interestingly, HLH‐30/TFEB recognizes and binds to so‐called CLEAR motifs (Grove et al., 2009; Lapierre et al., 2013; Settembre et al., 2013), which are found in numerous genes encoding mitochondrial proteins (Table S1). Accordingly, revisiting our previous expression analyses (Harvald et al., 2017) we find that genes encoding for mitochondrial proteins, for example, *cpt*‐*1*, *acs*‐*2*, and *hacd*‐*1* are downregulated in *hlh*‐*30* animals compared with wild type, while genes encoding for proteins involved in peroxisomal fatty acid metabolism, for example, acyl‐CoA oxidases are upregulated in *hlh*‐*30* animals compared with wild‐type animals (Figure S5). Our observations are perfectly aligned with recent observations by Weir et al. showing that remodeling of mitochondrial networks is required for AMPK‐ and dietary restriction‐mediated longevity, and that lifespan depends on both fatty acid oxidation and peroxisomal functions (Weir et al., 2017). Furthermore, although HLH‐30/TFEB primarily has been linked to lysosomal biogenesis, autophagy, and immune functions (Lapierre et al., 2013; Settembre et al., 2013; Visvikis et al., 2014), TFEB also exerts a global transcriptional control on glucose uptake and metabolism, fatty acid oxidation, oxidative phosphorylation, and mitochondrial biogenesis at the transcriptional level in rodents, among others via *Ppargc1α* and *Ppar1α* (Mansueto et al., 2017; Settembre et al., 2013).

The observation that fatty acid supplementation rescues lifespan of *hlh*‐*30* animals and extends longevity of wild‐type animals, suggests that mobilization of fatty acids from stored lipids is required for maintaining lifespan during starvation. Indeed, lipases like ATGL‐1, LIPL‐2, LIPL‐3, and LIPL‐5 are upregulated by dietary restriction and required for dietary restriction‐induced extension of lifespan (Buis et al., 2019; Lee et al., 2014; Murphy et al., 2019; Zaarur et al., 2019). Markedly, we recently found that loss of HLH‐30 diminishes expression of *atgl*‐*1*, *lipl*‐*2*, *lipl*‐*3*, and *lipl*‐*4* (Harvald et al., 2017). Here, we found that RNAi against *atgl*‐*1* shortens survival of wild‐type animals during starvation but not of *hlh*‐*30* animals. However, fatty acid supplementation extends lifespan of both wild‐type and *hlh*‐*30* animals, further supporting that mobilization of fatty acids from lipid stores provides metabolic energy to support organismal lifespan. Consistent with our findings, Macedo et al. recently reported that the abundance of certain cardiolipin species increases in response to dietary restriction (Macedo et al., 2019), which increased further upon loss of LIPL‐5.

Collectively, this study provides a comprehensive analysis of temporal starvation responses that combine both metabolomic and lipidomic analyses. Our data highlight the relevance of combining global profiling analyses to further understand how metabolism is regulated and how an organism adapts to nutritional changes. As exemplified by our comparison of the effect of starvation on metabolites and lipids in wild‐type and *hlh*‐*30* animals, the genetic tractability of *C. elegans* and combined with RNA interference, shows that the nematode system serves as an excellent framework to delineate conserved mechanisms whereby specific metabolic pathways regulate starvation responses.

## 4 EXPERIMENTAL PROCEDURES

### 4.1 *C. elegans* strains and maintenance

The wild‐type N2 Bristol and the *hlh*‐*30* mutant (tm1978, a kind gift from Dr. Marlene Hansen, Sandford‐Burnham Medical Research Institute) were used and cultivated under standard conditions and handled as described (Harvald et al., 2017).

### 4.2 Survival‐span assay

Worms were synchronized and grown until L4 stage on NGM seeded plates. For RNAi treatment, worms were transferred to plates containing IPTG seeded with the respective HT115 RNAi bacteria clone. At L4, worms were transferred to empty plates to induce starvation conditions. For supplementation with fatty acids, worms were transferred to plates containing either 40 µM lauric acid (C_12:0_) or palmitic acid (C_16:0_). 12 worms were placed on each plate and scored every day. A worm was scored dead when it was unresponsive to gentle prodding.

#### 4.2.1 Mitotracker staining and microscopy

Eggs from synchronized adults were grown until L4 stage on NGM seeded plates. Worms were stained as previously described (Ruiz et al. 2019). In short, worms were washed of plates with M9 buffer and washed three times. Mitotracker Deep Red FM (Cell signaling Technology) was dissolved in dimethylsulfoxide (DMSO) to a stock solution of 1 mM. Mitotracker was diluted to a 10 µM working solution in M9 buffer and worms were incubated for 2 h under rotation. Following incubation, worms were washed three times and transferred to NGM plates for scavenging. For microscopy, stained worms were mounted on 2% agarose pads containing 20 mM tetramizole and sealed with a cover slide. Mitochondria were visualized using a Nikon Ti‐2 Eclipse Widefield microscope and 60x water objective and images were processed using Image J.

#### 4.2.2 Fatty acid uptake

Synchronized L4 worms were washed thrice with M9 buffer and placed on empty NGM plates containing either 40 µM ^3^H‐lauric acid (^3^H‐C_12:0_, specific activity 2.9 µCi/µmol) or ^3^H‐palmitic acid (^3^H‐C_16:0_, specific activity 0.9 µCi/µmol)) added directly to the media. Both fatty acids were from Hartmann Analytic. After 24 h, 100 worms for each condition were picked and transferred to a scintillation vial containing 100 µl M9 buffer. Scintillation fluid (3 ml) was added, and vials were vortexed vigorously and left in the dark for 24 h before vials were subjected to scintillation counting in a MicroBeta^2^ System (Perkin Elmer). Subsequently, counts were converted to nmol fatty acid/100 worms.

### 4.3 Sample harvest for mass spectrometry

Worms were synchronized as described above and grown on NGM seeded plates until L4 stage and then transferred to empty NGM plates to induce starvation condition. Worms were harvested at specified time points by washing off plates with MS‐grade H_2_O and washed thrice. After the final wash, worms were incubated under rotation at RT for 20 min in H_2_O. Samples were spun down for 1 min at 1100 rpm/RT and supernatant aspirated. Samples were flash frozen in liquid nitrogen and stored at −80°C.

### 4.4 BUME extraction

Samples were extracted by using the BUME method as described (Lofgren et al., 2012) with minor modifications. Briefly, 50 µl ice‐cold methanol was added to each sample and transferred to beat‐beating tubes (NucleoSpin Bead Tubes Type A, Macherey Nagel). The samples were beat‐beaten for three times 10 s with 20 s pause in a Precellys Beat Beating system (Bertin Technologies). The additional Cryolys module was used with liquid nitrogen to prevent excessive heating of samples during disruption. 150 µl butanol and 200 µl heptane‐ethyl acetate (3:1) was added to each sample sequentially, which were then incubated for 1 h at 500 rpm/RT. 200 µl 1% acetic acid was added to each sample followed by centrifugation for 15 min at 17949 × g/4°C. The upper organic phase was transferred to a fresh Eppendorf tube, and the lower aqueous phase was re‐extracted by the addition of 200 µl heptane‐ethyl acetate followed by incubation and centrifugation as described above. The upper organic phase was transferred to the already obtained organic phase. The lower phase was transferred to a new Eppendorf tube and used for metabolomic analyses. Samples were evaporated to dryness and stored at −20°C. For lipidomics, samples were re‐dissolved in 50 µl 65% isopropanol/ 35% acetonitrile/ 5% H_2_O, vortexed and 40 µl were transferred to an autosampler vial. The remaining 10 µl were pooled to form a QC sample for the entire study. The precipitated proteins were used for determination of protein content using a Bicinchoninic Acid Protein Assay Kit (Sigma‐Aldrich).

For metabolomics, samples were re‐dissolved in 30 µl 1% formic acid, spun down for 5 min at 16,000 g / RT and 25 µl were transferred to HPLC vials. The remaining 5 µl were pooled to form a QC sample for the entire study.

### 4.5 Lipid analysis, data processing, and statistical analysis

Lipids were analyzed as previously described (Witting et al., 2014). Briefly, lipids were separated on a Waters Acquity UPLC (Waters) using a Waters Cortecs C18 column (150 mm x 2.1 mm ID, 1.6 µm particle size, Waters) and a linear gradient from 68% eluent A (40% H_2_O/60% acetonitrile, 10 mM ammonium formate, and 0.1% formic acid) to 97% eluent B (10% acetonitrile/90% isopropanol, 10 mM ammonium formate, and 0.1% formic acid). Mass spectrometric detection was performed using a Bruker maXis UHR‐ToF‐MS (Bruker Daltonic) in positive and negative ionization mode using data‐dependent acquisition to obtain MS^1^ and MS^2^ information. For every ten samples, a pooled QC was injected to check performance of the UPLC‐UHR‐ToF‐MS system and was used for normalization.

Raw data were processed with Genedata Expressionist for MS 12.0 (Genedata AG). Preprocessing steps included noise subtraction, m/z recalibration, chromatographic alignment and peak detection and grouping. Data were exported for Genedata Expressionist for MS 12.0 Analyst statistical analysis software and as.xlxs for further investigation. Maximum peak intensities were used for statistical analysis, and data were normalized on the protein content of the sample and an intensity drift normalization based on QC samples was used to normalize for the acquisition sequence.

Lipids were putatively annotated on the MS^1^ level using an in‐house developed database for *C. elegans* lipids and bulk composition from LipidMaps (O'Donnell et al., 2019), when available. MS2 data were extracted using Bruker Data Analysis 5.0 (Bruker Daltonics) and read into R using the MSnbase package (Gatto & Lilley, 2012). Matching against an *in*‐*silico* database of *C. elegans* lipids and LipidBlast was performed using the masstrixR package (Kind et al., 2013; Witting unpublished, https://github.com/michaelwitting/masstrixR), and only hits with a forward and reverse matching score >0.75 were considered. Lipids with a *p*‐value <.05 and a fold‐change of >2 or <0.5 were considered to be significantly changed. Annotations of interesting biological peaks were manually verified and corrected if necessary.

### 4.6 Metabolomics analysis and data processing of BUME extraction

5 µl were injected using a Vanquish Horizon UPLC (Thermo Fisher Scientific) and compounds separated on a Zorbax Eclipse Plus C18 guard (2.1 × 50 mm and 1.8 μm particle size, Agilent Technologies), and an analytical column (2.1 × 150 mm and 1.8 μm particle size, Agilent Technologies) kept at 40°C. The analytes were eluted using a flow rate of 400 μl/min and the following composition of eluent A (0.1% formic acid) and eluent B (0.1% formic acid, acetonitrile) solvents: 3% B from 0 to 1.5 min, 3% to 40% B from 1.5 to 4.5 min, 40% to 95% B from 4.5 to 7.5 min, 95% B from 7.5 to 10.1 min and 95% to 3% B from 10.1 to 10.5 min before equilibration for 3.5 min with the initial conditions. Using a 6‐port valve and a secondary pump, the guard wash backflushed from 9 to 10 min with a flow of 1 ml/min with 95% B. The flow from the UPLC was coupled to a Q Exactive HF mass spectrometer (Thermo Fisher Scientific) for mass spectrometric analysis in both positive and negative ion mode using the following general settings for MS1 mode: resolution: 120,000, AGC target: 3e6, maximum injection time: 200 ms, scan range 65–975 m/z, and lock mass: 391.28429/112.98563 (pos/neg mode). For compound fragmentation, MS1/ddMS2 mode was used with the following general settings: resolution: 60,000/15,000, AGC target: 1e6/1e5, maximum injection time: 50/100 ms, scan range 65 to 975 m/z, loop count: 5, isolation with: 2 m/z and normalized collision energy: 35/38 (pos/neg). The generated pooled sample was used for quality control (QC) and compound fragmentation. Samples were analyzed in randomized order with a QC sample injected every 9th run. A MS2 inclusion list was generated from the blanks and the last equilibration runs using Compound Discover v. 3.0 (Thermo Fisher Scientific) to ensure fragmentation of the later extracted features. The features found in the blank were removed from the inclusion list if they were not >5 x more abundant in the QC samples.

Raw data were processed with MzMine (v 2.42; Pluskal et al., 2010). In brief, the following modules were used: Mass detection, ADAP chromatogram builder, ADAP deconvolution, Join aligner, Isotopic peak grouper, Gap filling (same RT and m/z range), and identification in local spectra database search; all with 5 ppm mass tolerance and 0.25 RT tolerance when possible. Final peaklist included features found in at least 60% of the samples, which had at least 2 peaks in an isotope pattern. Compounds were annotated at metabolomics standards initiative (MSI; Salek et al., 2013) level 2 using local MS/MS spectra databases of National Institute of Standards and Technology 17 (NIST17) and MassBank of North America (MoNA). Moreover, the MS/MS data were also searched in MzCloud using Compound Discoverer for additional annotations at MSI level 2. MSI level 3 annotation was achieved using SIRIUS (Duhrkop et al., 2019) before lastly MSI level 4 annotation by searching in human metabolite database (Wishart et al., 2018). After compound annotation, the datasets were corrected for signal drift using the R package statTarget (Luan et al., 2018). Metabolites with a *p*‐value <.05 and a fold‐change of >2 or <0.5 were considered to be significantly changed.

### 4.7 MeOH/ACN/H_2_O extraction of metabolites

Samples were harvested as described above (2000 worms per. sample in biological triplicates) and flash frozen to be stored at −80°C. Samples were thawed on ice and 200 µl ice‐cold extraction solvent (50% methanol/30% acetonitrile/20% H_2_O, MS‐grade) was added to each sample followed by vigorous vortexing. Samples were sonicated in an ice bath for 10 x 30 s at high speed. Samples were incubated in a Thermoshaker for 30 min at 1.200 rpm/4°C, vortexed vigorously and spun down for 10 min at 12,000 g/4°C. The supernatant was transferred to fresh Eppendorf tubes, lyophilized and stored at −20°C. Before injection samples were re‐dissolved in 30 µl 0.1% formic acid, spun down for 5 min at 16,000 g/RT and supernatant transferred to HPLC vials.

### 4.8 Metabolomics analysis and data processing of MeOH/ACN/H_2_O extracted metabolites

Metabolites were analyzed according to (Sustarsic et al., 2018) with minor alterations to the gradient. In brief, metabolites were analyzed with LC–MS using reverse phase (RP) separation. 5 μl were injected using an Agilent 1290 Infinity HPLC system (Agilent Technologies) equipped with an Agilent Zorbax Eclipse Plus C18 column (2.1 x 150 mm, 1.8 μm, Agilent Technologies) with a 50 mm guard‐column, both kept at 40°C. The analytes were eluted using a flow rate of 300 µl/min and solvent with the following eluent composition: eluent A (0.1% formic acid, H_2_O) and eluent B (0.1% formic acid, acetonitrile): 97% A from 0 to 8 min, 60% A from 8 to 12 min, 10% A from 12 to 15 min before equilibration with initial conditions for 3 min. The HPLC flow was coupled to an Agilent 6530 quadrupole time of flight (Q‐TOF) mass spectrometer scanning from 70 to 1000 m/z. A pooled sample was generated for compound fragmentation, and all samples were analyzed in both positive and negative ion mode. Samples were run in all‐ion fragmentation mode with collision energy of 20 V, in order to produce fragments for identification of metabolites. Libraries of metabolites with retention time (RT) were constructed using Agilent MassHunter PCDL Manager. The identification of each compound was based on exact mass, RT, and/or comparison of fragments with the Metlin MS/MS database (https://metlin.scripps.edu). Chromatograms for all compounds were extracted and quantified using Agilent Profinder using a mass tolerance of 20 ppm and a retention time tolerance of 0.1 min.

### 4.9 β‐oxidation assay

The method for measuring fatty acid oxidation was applied as described previously (Elle et al., 2012).

## CONFLICT OF INTEREST

None declared.

## AUTHOR CONTRIBUTIONS

K.B.D., M.W., and N.J.F. designed the experiments. K.B.D., M.W., and N.J.F. wrote the manuscript. K.B.D., E.B.H., J.F.H., M.W., and N.J.F. performed the experiments and analyzed the data.

## Supporting information

Fig S1‐S5

Table S1‐S3

## Data Availability

Data and R scripts have been deposited and are available at www.mendeley.com; http://dx.doi.org/10.17632/2r27v9yjp5.1
