# Supplementary material for: HLH‐30‐dependent rewiring of metabolism during starvation in C. elegans
Source: Aging Cell. 2021 Mar 16;20(4):e13342. doi: 10.1111/acel.13342 (PMC8045935; doi:10.1111/acel.13342)
Supplement: Supplementary file 2 — Table S1‐S3 [file ACEL-20-e13342-s001.pdf]

Figures and Tables

Strain	FA treat-ment	FA treated survival -span (d)	FA treated survival-span (d)*	No. of treated animals	Control survival-span (d)	Control survival-span (d)*	No. of Control animals	Max survival-span (d)	*p*-value versus Control
**N2 Bristol** (WT)	$C_{12:0}$	11 (45.4%)	10.75 (36.5%)	93/120	6	6.82	91/120	16/10	<0.0001
	$C_{12:0}$	10 (20%)	9.96 (26.9%)	85/120	8	7.28	93/120	17/10	<0.0001
	$C_{12:0}$	13 (53.8%)	12.03 (49.6%)	95/120	6	6.06	93/120	16/10	<0.0001
	$C_{16:0}$	8 (25%)	7.74 (21.7%)	109/120	6	6.06	93/120	12/10	<0.0001
	$C_{16:0}$	9 (11.1%)	8.53 (13.0%)	94/120	8	7.42	97/120	12/10	<0.0001
	$C_{16:0}$	9 (11.1%)	7.89 (5.70%)	106/120	8	7.44	87/120	12/10	0.0045
**JIN1375** (*hlh-30*)	$C_{12:0}$	10 (40%)	8.79 (37.8%)	103/120	6	5.46	81/120	15/8	<0.0001
	$C_{12:0}$	9 (44.4%)	9.43 (46.0%)	103/120	5	5.09	89/120	16/7	<0.0001
	$C_{12:0}$	13 (61.5%)	10.78 (62.2%)	104/120	5	4.07	88/120	15/7	<0.0001
	$C_{16:0}$	8 (37.5%)	7.43 (45.2%)	98/120	5	4.07	88/120	9/7	<0.0001
	$C_{16:0}$	8 (50%)	7.51 (43.6%)	98/120	4	4.23	90/120	10/6	<0.0001
	$C_{16:0}$	9 (44.4%)	7.29 (37.4%)	102/120	5	4.56	89/120	10/7	<0.0001

Supplementary Table S2. Summary of survival-span statistics. Median, mean and maximum survival and statistical significance were calculated for each experiment. *Mean survival lifespan. *p*-values were based on median survival lifespan.

Strain	RNAi treat-ment	FA treat-ment	RNAi / FA treated survival-span (d)	RNAi / FA treated survival-span (d)*	No. of treated animals	Control survival-span (d)	Control survival-span (d)*	No. of Control animals	Max survival -span (d)	*p*-value versus Control
**N2 Bristol** (WT)	*prx-5* (JA)	none	8 (0%)	7 (-3.28%)	89/120	8	7.23	89/120	11/10	0.4624
	prx-5 (JA)	none	8 (-12.5%)	7.36 (-2.58%)	88/120	9	7.55	83/120	10/10	0.2838
	prx-5 (JA)	none	9 (11.11%)	7 (-1%)	85/120	8	7.07	84/120	10/10	0.7177
	atgl-1 (JA)	none	6 (-33.32%)	5.21 (-38.77%)	88/120	8	7.23	89/120	8/10	<0.0001
	atgl-1 (JA)	none	7 (-28.57%)	6.47 (-16.69%)	88/120	9	7.55	83/120	8/10	< 0.0001
	atgl-1 (JA)	none	5 (-60%)	4.75 (-48.84%)	84/120	8	7.07	84/120	8/10	< 0.0001
	cpt-1 (JA)	none	3 (-166.66%)	3.38 (-106.21%)	83/120	8	6.97	86/120	5/10	< 0.0001
	prx-5 (JA)	$C_{12:0}$	12 (0%)	9.92 (-6.04%)	88/120	12	10.52	87/120	15/15	0.3020
	prx-5 (JA)	$C_{12:0}$	12 (8.33%)	10.17 (-3.34%)	84/120	11	10.51	86/120	15/15	0.8463
	prx-5 (JA)	$C_{12:0}$	10 (-10%)	9.46 (-2.21%)	105/120	11	9.67	107/120	15/15	0.2236
	atgl-1 (JA)	$C_{12:0}$	11 (-9.09%)	9.57 (-9.92%)	89/120	12	10.52	87/120	15/15	0.1780
	atgl-1 (JA)	$C_{12:0}$	12 (8.33%)	10.59 (0.75%)	88/120	11	10.51	86/120	15/15	0.8616
	atgl-1 (JA)	$C_{12:0}$	12 (8.33%)	9.76 (0.92%)	95/120	11	9.67	107/120	15/15	0.9674
	cpt-1 (JA)	$C_{12:0}$	9 (-11.11%)	8.11 (-14.05%)	102/120	10	9.25	98/120	15/15	0.2773
	prx-5 (JA)	$C_{16:0}$	8.5 (-41.17%)	8.61 (-12.07%)	104/120	12	9.65	89/120	14/14	0.0228
	prx-5 (JA)	$C_{16:0}$	9 (-22.22%)	9.17 (-4.14%)	91/120	11	9.55	85/120	14/14	0.462
	prx-5 (JA)	$C_{16:0}$	10 (-5%)	9 (-2.77%)	101/120	10.5	9.25	110/120	14/14	0.3309
	atgl-1 (JA)	$C_{16:0}$	11 (-9.09%)	9.78 (1.32%)	87/120	12	9.65	89/120	14/14	0.5313
	atgl-1 (JA)	$C_{16:0}$	12 (8.33%)	10.18 (6.18%)	89/120	11	9.55	85/120	14/14	0.1805
	atgl-1 (JA)	$C_{16:0}$	11.5 (8.33%)	9.38 (1.38%)	102/120	10.5	9.25	110/120	14/14	0.6918
	cpt-1 (JA)	$C_{16:0}$	3 (-200%)	5.48 (-53.28%)	104/120	9	8.4	97/120	12/15	< 0.0001

Supplementary Table S3A. Summary of survival-span statistics for wildtype animals. Median, mean and maximum survival and statistical significance were calculated for each experiment. *Mean survival lifespan. *p*-values were based on median survival lifespan.

Strain	RNAi treat ment	FA treat-ment	RNAi / FA treated survival-span (d)	RNAi / FA treated survival-span (d)*	No. of treated animals	Control survival-span (d)	Control survival-span (d)*	No. of Control animals	Max survival -span (d)	*p*-value versus Control
**JIN1375** (*hlh-30*)	*prx-5* (JA)	none	3 (-33.33%)	3.16 (-25.63%)	86/120	4	3.97	85/120	6/6	< 0.0001
	prx-5 (JA)	none	3 (-33.33%)	3.09 (-22.33%)	93/120	4	3.78	88/120	5/6	< 0.0001
	prx-5 (JA)	none	3 (-33.33%)	2.98 (-25.50%)	84/120	4	3.74	85/120	5/6	< 0.0001
	atgl-1 (JA)	none	4 (0%)	3.56 (-11.51%)	90/120	4	3.97	85/120	6/6	0.0032
	atgl-1 (JA)	none	4 (0%)	3.5 (-8%)	94/120	4	3.78	88/120	5/6	0.0018
	atgl-1 (JA)	none	3 (-33.33%)	3.15 (-18.73%)	83/120	4	3.74	85/120	5/6	0.0004
	cpt-1 (JA)	none	4 (0%)	3.74 (3.20%)	87/120	4	3.62	85/120	6/6	0.6441
	prx-5 (JA)	$C_{12:0}$	4 (75%)	4.06 (-88.91%)	87/120	7	7.67	84/120	6/14	< 0.0001
	prx-5 (JA)	$C_{12:0}$	3 (-233.33%)	3.3 (-173.63%)	86/120	10	9.03	91/120	5/14	< 0.0001
	prx-5 (JA)	$C_{12:0}$	3 (-233.33%)	3.1 (184.83%)	94/120	10	8.83	112/120	5/15	< 0.0001
	atgl-1 (JA)	$C_{12:0}$	6 (-16.66%)	5.5 (-39.45%)	88/120	7	7.67	84/120	10/14	< 0.0001
	atgl-1 (JA)	$C_{12:0}$	9 (-11.11%)	7.29 (-23.86%)	87/120	10	9.03	91/120	10/14	< 0.0001
	atgl-1 (JA)	$C_{12:0}$	11.5 (13.04%)	9.33 (5.35%)	98/120	10	8.83	112/120	14/15	0.4269
	cpt-1 (JA)	$C_{12:0}$	9 (0%)	8.37 (0%)	101/120	9	8.37	100/120	15/15	0.4095
	prx-5 (JA)	$C_{16:0}$	4 (-75%)	4.33 (-51.27%)	100/120	7	6.55	90/120	6/10	< 0.0001
	prx-5 (JA)	$C_{16:0}$	3 (-200%)	3.19 (-118.49%)	84/120	9	6.97	86/120	5/10	< 0.0001
	prx-5 (JA)	$C_{16:0}$	3 (-200%)	2.9 (-189.31%)	102/120	9	8.39	103/120	5/12	< 0.0001
	atgl-1 (JA)	$C_{16:0}$	6 (-16.6%)	6.03 (-8.62%)	91/120	7	6.55	90/120	10/11	0.0068
	atgl-1 (JA)	$C_{16:0}$	9 (0%)	7.34 (5.04%)	89/120	9	6.97	86/120	10/10	0.4547
	atgl-1 (JA)	$C_{16:0}$	11 (18.18%)	8.48 (1.06%)	94/120	9	8.39	103/120	13/14	0.1734
	cpt-1 (JA)	$C_{16:0}$	6.5 (-7.69%)	6.05 (-3.63%)	110/120	7	6.27	105/120	10/10	0.0637

Supplementary Table S3B. Summary of survival-span statistics for *hlh-30* mutant animals. Median, mean and maximum survival and statistical significance were calculated for each experiment. *Mean survival lifespan. *p*-values were based on median survival lifespan.
